# Nanostructured Flame-Retardant Layer-by-Layer Architectures for Cotton Fabrics: The Current State of the Art and Perspectives

**DOI:** 10.3390/nano14100858

**Published:** 2024-05-15

**Authors:** Giulio Malucelli

**Affiliations:** 1 Department of Applied Science and Technology, Politecnico di Torino, Viale Teresa Michel 5, 15121 Alessandria, Italy; giulio.malucelli@polito.it; Tel.: +39-0131229369; 2 Consorzio Interuniversitario Nazionale per la Scienza e Tecnologia dei Materiali (INSTM), Via G. Giusti 9, 50121 Florence, Italy

**Keywords:** flame retardancy, layer-by-layer method, nano-objects, cotton, flammability, combustion behavior, multifunctionality, durability

## Abstract

Nowadays, nanotechnology represents a well-established approach, suitable for designing, producing, and applying materials to a broad range of advanced sectors. In this context, the use of well-suited “nano” approaches accounted for a big step forward in conferring optimized flame-retardant features to such a cellulosic textile material as cotton, considering its high ease of flammability, yearly production, and extended use. Being a surface-localized phenomenon, the flammability of cotton can be quite simply and effectively controlled by tailoring its surface through the deposition of nano-objects, capable of slowing down the heat and mass transfer from and to the textile surroundings, which accounts for flame fueling and possibly interacting with the propagating radicals in the gas phase. In this context, the layer-by-layer (LbL) approach has definitively demonstrated its reliability and effectiveness in providing cotton with enhanced flame-retardant features, through the formation of fully inorganic or hybrid organic/inorganic nanostructured assemblies on the fabric surface. Therefore, the present work aims to summarize the current state of the art related to the use of nanostructured LbL architectures for cotton flame retardancy, offering an overview of the latest research outcomes that often highlight the multifunctional character of the deposited assemblies and discussing the current limitations and some perspectives.

## 1. Introduction

The development and implementation of surface engineering strategies have paved the way for the progress in the design of advanced (multi)functional materials: indeed, the surface of any material “rules” the interactions taking place between the material itself and the surrounding environment. Therefore, providing the surface of any substrate with specific functionalities (even more than one at the same time) makes the material suitable for applications that would have never been envisaged without that particular surface treatment. Additionally, these newly conferred characteristics do not modify the overall bulk properties of the treated substrate, hence (i) avoiding the use of high amounts of additives/modifiers/fillers that are usually diluted in the material’s bulk (thus providing a limited effect), (ii) determining a negligible impact on those properties that do not require any modification, and, finally, (iii) focusing on the most critical area, i.e., the material’s surface [1,2,3].

**Figure 1:**
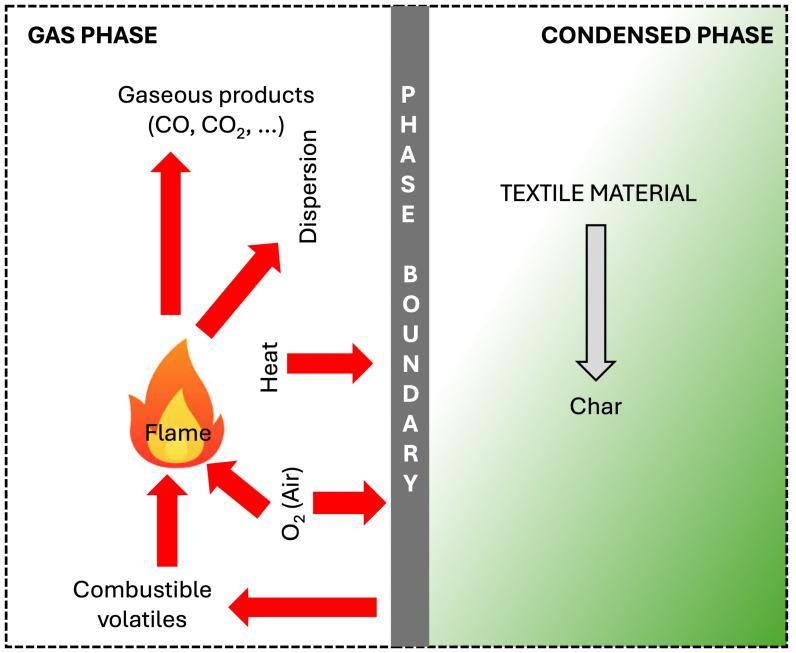
A scheme of the flaming combustion cycle for a textile material.

The surface engineering approach has successfully been exploited for limiting or even blocking the easy flammability of both natural and synthetic textile materials. Indeed, if not inherently flame-retarded, fibers and fabrics easily burn when put in contact with a direct flame or exposed to an irradiative heat flux. Therefore, they require the use of flame retardants (FRs) [4,5,6], i.e., additives capable of lowering the fire risk by either stopping the ignition or slowing down the flame spread rate during a combustion occasion. FRs may exhibit a prevalent action in the condensed phase or the gas phase, although they can be active in both: as depicted in Figure 1, upon the application of a direct flame or a heat flux, the textile material begins to degrade, giving rise to the formation of gaseous combustible products that, in turn, mix with air and contribute to fueling the flame, also producing fully (i.e., CO_2_, water) or partially (CO) oxidized products. The combustion cycle becomes self-sustainable when the part of the heat generated by the highly exothermic process transferred to the textile material is enough. A flame retardant active in the condensed phase limits the heat and mass transfer phenomena that recurrently feed the flammable degradation products (responsible for the fire spread), through the formation of a surface protective (and often thermally insulating) layer, also capable of lowering the extent of thermal feedback that supplies the fire spread [7,8,9]. Conversely, the involved mechanisms, through which a flame retardant can be active in the gas phase, refer to (i) the radical scavenging of the unstable radicals (such as H· and OH·), which are responsible for the flame spread, by less reactive radicals provided by the decomposition of the flame retardants, or (ii) the physical attenuation of the combustible gases through the endothermic release of such non-flammable gaseous species as water vapor, nitrogen, and carbon dioxide [10,11].

Specifically regarding flame retardancy, several surface engineering methods have been applied to flammable polymeric materials (i.e., bulk polymers, textiles, foams, and composites), leading to a significant increase in the resistance of the treated polymers toward the application of a direct flame or the exposure to an irradiative heat flux [12,13,14].

Among the different developed strategies for enhancing the flame retardancy of textiles (namely, plasma-aided treatments [15,16], nanoparticle absorption [17,18], sol–gel processes [19,20], and back-coating [21,22]), the layer-by-layer strategy is definitively emerging as one of the most effective and promising top-down techniques, also considering the possibility of conferring multifunctional features to various types of polymeric substrates.

Iler invented LbL in 1966, providing a proof of concept of the possibility of creating nanostructured assemblies of colloidal particles by exploiting a molecularly controlled method [23]. Then, LbL was somehow abandoned until 1991, when Decher and Hong discovered a practical and feasible procedure for producing ultrathin multi-layered architectures made of alternated amphiphilic polyanionic/polycationic layers [24].

Nowadays, the importance of this surface-engineered approach is still growing, stimulating several research groups of both academia and industry to pave the way toward new potential applications, thanks to the peculiar features of layer-by-layer assemblies, namely as follows: the wide availability of layers’ constituents of different chemical structures, composition, and various shape/aspect ratios, which range from polyelectrolytes to “objects” (micro and nano) and even to biomolecules and biomacromolecules; the effectiveness of a few layers in conferring the envisaged properties to the treated substrate; the possibility of using quite low environmental impact processes; the mild conditions adopted for its application (usually room temperature and atmospheric pressure); and the ease of application (by dipping or spraying) of the overall method [25,26,27,28].

**Figure 2:**
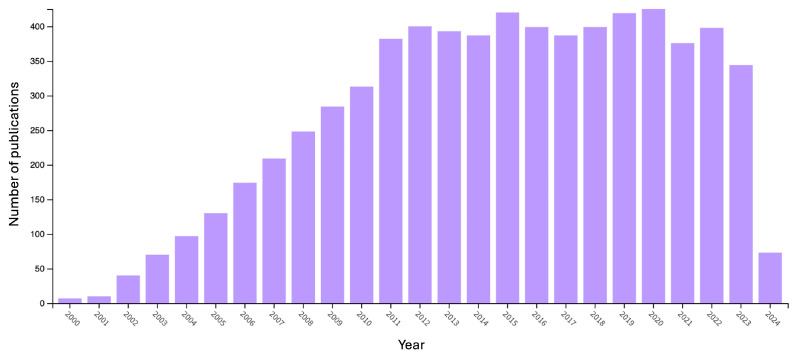
The number of publications (from 2000 to 2024) in peer-reviewed journals, dealing with “Layer-by-Layer” (data collected from the Web of Science^TM^ database, www.webofscience.com, accessed on 14 April 2024).

All these research efforts on this topic are clearly witnessed by the remarkably increasing number of articles published in the last 15 to 20 years (Figure 2). Further, the extended research carried out so far is well documented by several reviews that appeared in the scientific literature [29,30,31,32,33,34,35,36,37,38,39].

The present review aims to discuss the current state of the art related to the use of LbL architectures made of nanostructured components for conferring flame retardancy to cotton fabrics. Indeed, cotton, i.e., the natural seed fiber that derives from the cotton plant, is the second most common fiber used today after polyester, making up 26–35% of the overall textile market [40]; additionally, it is the most widely used natural fiber in the textile industry because of its numerous advantages (i.e., natural abundance, softness, comfort, hydrophilicity, air permeability, and biodegradability, among others), and therefore it is widely used in residential, industrial, medical, and other fields and accounts for 30–50% of the total fibers’ utilization [41]. However, when exposed to a flame or a heat flux, it burns vigorously and therefore needs to be protected [42].

First, to meet the perspectives of readers who are not familiar with the proposed topic, the fundamentals of the LbL technique will be elucidated. Then, an overview of the continuous progress (from the beginning) on the design and implementation of flame-retardant nanostructured LbL architectures applied to cotton fabrics will be summarized, finally focusing on the very latest research outcomes. Lastly, the current limitations will be discussed, together with some perspectives envisaging possible further research trends for this intriguing surface-engineered method.

## 2. Basics of the Layer-by-Layer Methodology

**Figure 3:**
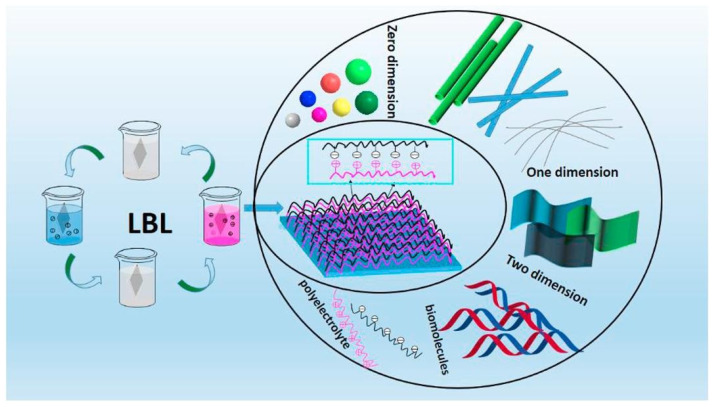
A scheme of the LbL dipping process: the substrate is sequentially immersed into baths containing dilute aqueous solutions/suspensions of the “objects” (particles, rods/tubes, sheets/lamellae, polyelectrolytes, biomacromolecules) to deposit. Reprinted with permission from [27]. Copyright 2018, Elsevier.

As mentioned in the Introduction paragraph, creating an LbL assembly on a substrate usually requires the alternate deposition of oppositely electrically charged layers, made of polyelectrolytes, micro- or nanoparticles, rods/tubes, sheets/lamellae, or bio(macro)molecules. However, instead of exploiting electrostatic interactions among the deposited layers, it is possible to employ other types of interactions, namely as follows: hydrogen bonding [43,44], covalent bonding [45,46], and donor/acceptor exchanges [47,48]. Figure 3 depicts a general schematization of the LbL methodology.

**Figure 4:**
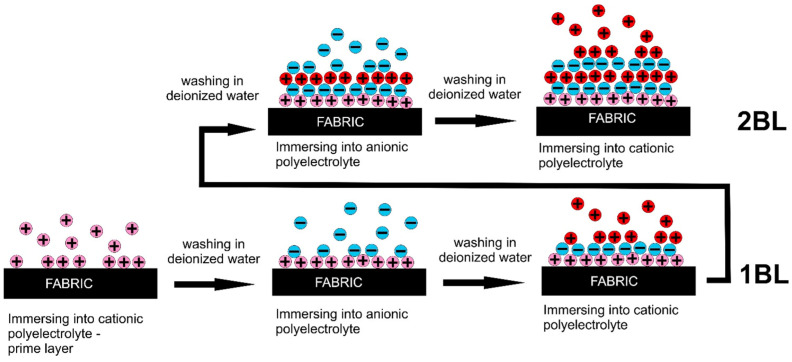
A closer look at the dipping-assisted LbL process employing polyelectrolyte baths: the use of a primer layer. Reprinted from [49] under BB-CY License.

Because of the dipping in the treating baths, the components of the LbL assemblies are successively adsorbed as nano-layers on the surface of the polymer substrate, leading to the formation of a complex multi-layered assembly made of the repetition of bi-, tri-, or quad-layers, according to the sequence adopted for each dipping step and exploiting the reversal of the surface charges. Quite often, the deposition of a first layer (acting as a primer) is carried out, aiming to improve the adhesion of the fabric with the successive layers of the assembly (Figure 4).

Alongside the “traditional” dipping method, spray-assisted techniques have also been designed and implemented: unlike dipping, which is more suitable for lab-scale treatments, spraying is preferable for pilot/industrial plants. Every single dipping or spraying step has a variable duration, which is strictly related to the type of substrate to treat, the constituents of the deposited layers, and possible rinsing and drying steps that may take place in between every dipping stage. It is worth noticing that rinsing and drying steps are not mandatory: rinsing is usually suggested not only to remove the excess of the inappropriately adhered constituents of the layers but also to prevent contamination that may happen during two consecutive dipping steps. The construction of the LbL assembly proceeds until the intended number of layers has been deposited on the substrate: in this context, ten to fifty (or even more) layers can be placed on the polymer substrate, depending on the design of the LbL architecture. Additionally, several experimental parameters may significantly affect the structure, morphology, and final performance of the layer-by-layer assemblies, namely as follows: adsorption time [50,51], the pH of the solutions/suspensions [52], ionic strength [53,54], temperature [55,56], and the structure/conformation/molecular weight of the employed polyelectrolytes [57,58,59].

## 3. Nanostructured LbL Assemblies for Flame-Retarded Cotton: The Origins and Early Research Progress

**Figure 5:**
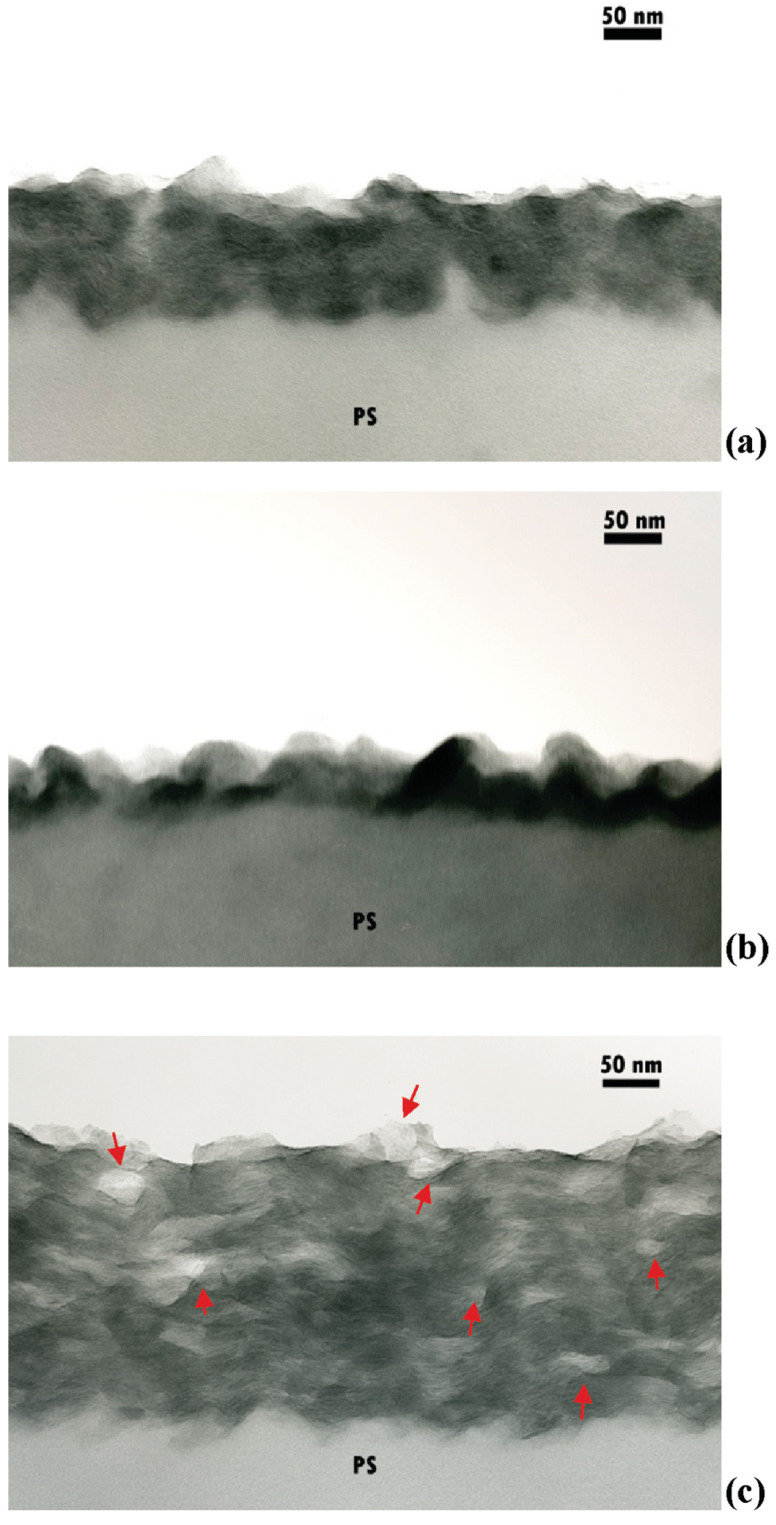
TEM cross-sectional images of assemblies (30 bi-layers) made with Laponite and branched poly(ethyleneimine) at pH 10 (**a**) and pH 8 (**b**) and with branched poly(ethyleneimine) and Laponite at pH 6 (**c**); all the assemblies were grown on a polystyrene (PS) film. Several light-colored round or elliptical areas appeared in the lateral view of the cross-section (**c**) (highlighted by red arrows), which correspond to the size of Laponite platelets tilted on their sides. Reprinted with permission from [60]. Copyright 2009, American Chemical Society.

One of the first pioneering papers demonstrating the potential of nanostructured flame-retardant LbL assemblies for cotton fabrics comes from Grunlan’s group [60], who designed bi-layered assemblies (up to 30) made of branched poly(ethyleneimine) and nanoclay platelets (namely, synthetic Laponite) and investigated the effect of pH on the growth, mechanical behavior, and flammability of the treated cellulosic fabrics. Each deposited layer ranged from 0.5 to about 5 nm, depending on the nanoclay content (Figure 5). A sort of cobblestone path was observed in atomic force microscopy analyses, due to the morphology of the obtained assemblies that accounted for the increased thermo-oxidative stability of cotton, as well as increased hardness and enhanced residues at the end of vertical flame spread tests (though the LbL-treated fabrics were not self-extinguishing), due to the thermal shielding effect exerted by the deposited architectures.

Then, the bi-layer structure was modified, by replacing Laponite with montmorillonite [61]: in this case, not only the pH of the dipping bath of the branched poly(ethyleneimine) (i.e., 7 and 10) but also the nanoclay content (0.2 and 1 wt.%) and the number of deposited bi-layers (namely, 5 and 20) were changed. As revealed by vertical flame spread tests, the cotton fabrics treated with 20 bi-layers (1 wt.% nanoclay loading and operating with a bath at pH = 10) accounted for a remarkable increase in the residues at the end of the tests, hence indicating an extended char-forming character of the deposited assembly. Further, this latter was responsible for an important decrease in heat release capacity (HRC) with respect to the untreated cellulosic substrate (about −19%), as revealed by pyrolysis–combustion flow calorimetry tests. Once again, the pH of the branched poly(ethyleneimine) bath was found to be a key parameter in defining the compactness, morphology, and crosslinking density of the resulting LbL architectures, because of the strict relationship between the pH of the dipping bath and the ionization degree of the employed polyelectrolyte.

In a further research effort, cotton fabrics were coated with a fully inorganic nanostructured assembly made of positively charged alumina-coated silica particles (10 nm size) and negatively charged silica particles (8 or 27 nm size), utilizing a dipping-assisted process [62]. The deposition of 20 bi-layers on the fabrics allowed for achieving self-extinction in vertical flame spread tests. The thermal protection exerted by this ceramic assembly was further confirmed by pyrolysis–combustion flow calorimetry tests, which evidenced a 14% decrease in the peak of the heat release rate.

**Figure 6:**
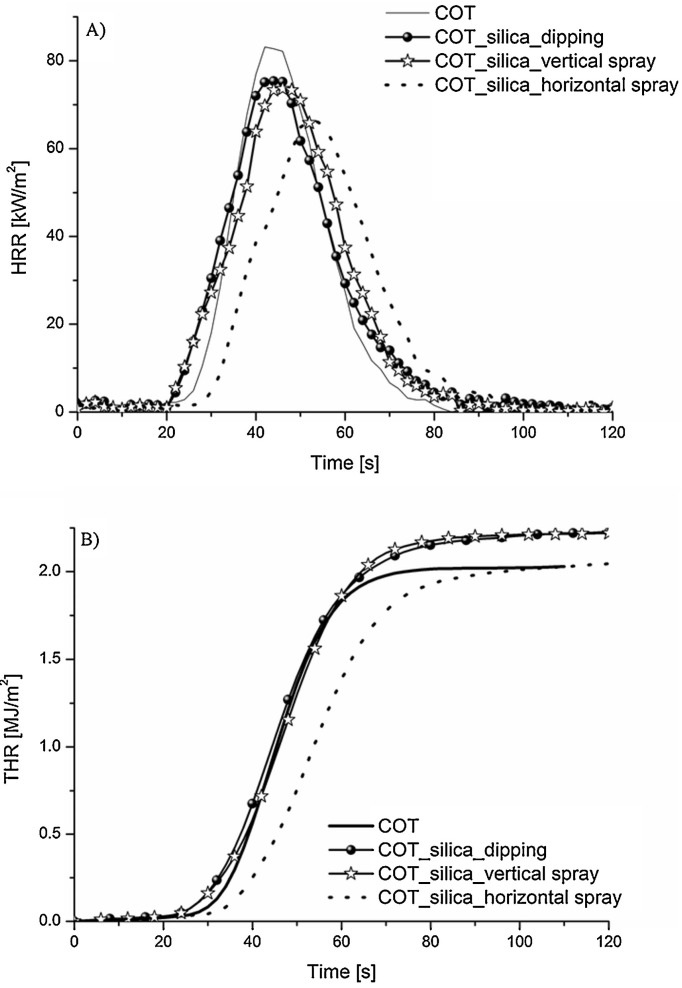
(**A**) Heat release rate (HRR) and (**B**) total heat release (THR) curves for untreated cotton (COT) and the LbL-treated fabrics through dipping, vertical, and horizontal spray-assisted processes. Reprinted with permission from [63]. Copyright 2013, Elsevier.

The same assembly was also deposited on cotton, replacing LbL dipping with vertical or horizontal spraying [63]. The horizontal spraying of five bi-layers showed the best performance in forced-combustion tests (irradiative heat flux: 35 kW/m^2^), with an increase in the time to ignition (+21%) and a decrease in both the peak of the heat release rate (−20%) and Total Smoke Release (−16%), together with a shift in the total heat release toward longer times (Figure 6).

An interesting hybrid assembly made of an intumescent polyacrylamide (derived from the copolymerization reaction of acrylamide with N1-(5,5-dimethyl-1,3,2-dioxaphosphinyl-2-yl)-acrylamide) and graphene oxide nanosheets was proposed by Huang and co-workers [64], who deposited 5 or 20 bi-layers on cotton fabrics. As assessed by forced-combustion tests (performed at 35 kW/m^2^ irradiative heat flux), the deposition of 20 bi-layers accounted for about a 22 and 50% decrease in the total heat release and peak of the heat release rate, respectively, as well as for about a 36% increase in the time to ignition compared with the untreated cellulosic substrate, hence indicating a significant flame retardant action in condensed phase provided by the LbL assembly.

Pursuing this research, the same group [65] designed bi-layered assemblies combining amino-functionalized montmorillonite nanoplatelets with a copolymer of acrylic acid and N-(2-(5,5-dimethyl-1,3,2-dioxaphosphinyl-2-ylamino)-ethylacetamide-2-propenyl acid. As assessed by forced-combustion tests (irradiative heat flux: 35 kW/m^2^), compared with the untreated counterparts, the cotton fabrics treated with 20 bi-layers exhibited a 47 and 18% decrease in the peak of the heat release rate and total heat release, respectively, as well as an increased time to ignition (i.e., 14 s longer). These findings were ascribed to the concurrent thermal protection exerted by the montmorillonite nanoplatelets and the intumescent character of the copolymer layers.

Polyhedral oligomeric silsesquioxanes (POSSs) are well-defined 3D organic/inorganic hybrid molecules, based on a silica-like core covered with an organic shell; their peculiar structures can easily be integrated with different polymers through chemical crosslinking, physical blending, and covalent grafting among others [66,67]. Li and co-workers [68] demonstrated the feasibility of using POSS cages as cationized and anionized constituents of fully inorganic bi-layered flame-retardant assemblies on cotton fabrics. In particular, 5, 10, and 20 bi-layers of OctaAmmonium POSSs (positively charged) and OctaTetramethylAmmonium POSSs (negatively charged) were alternately deposited on the cellulosic substrate: as assessed by pyrolysis–combustion flow calorimetry tests, 20 bi-layers decreased both the peak of the heat release rate (by about 11%) and the total heat release (by around 17%) compared to the untreated fabric. In addition, although it was not possible to reach self-extinction, the deposited LbL assemblies were responsible for a decrease in the afterglow times, as revealed by vertical flame spread tests.

**Figure 7:**
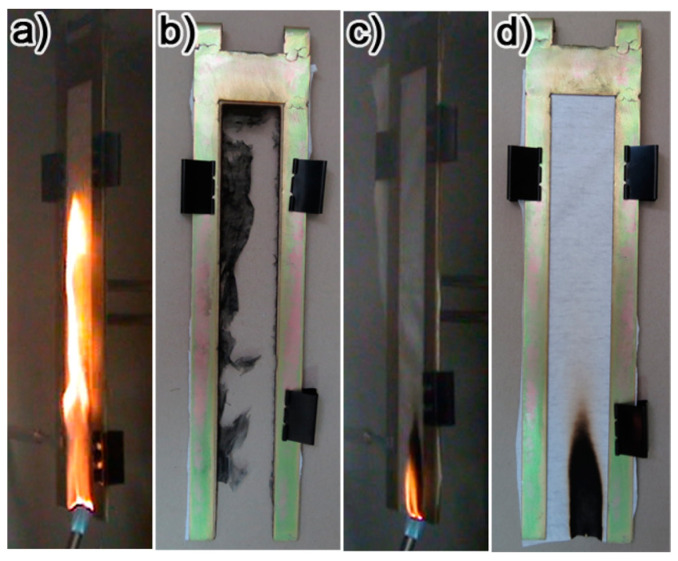
The results from vertical flame spread tests: uncoated fabric recorded at 4 s after ignition (**a**); uncoated fabric at the end of the test (**b**); cotton fabric coated with an intumescent tri-layer made of branched poly(ethylenimine), ammonium polyphosphate, and fluorinated-decyl polyhedral oligomeric silsesquioxane recorded at 4 s after ignition (**c**); and cotton fabric coated with an intumescent tri-layer made of branched poly(ethylenimine), ammonium polyphosphate, and fluorinated-decyl polyhedral oligomeric silsesquioxane at the end of the test (**d**). Reprinted with permission from [69]. Copyright 2015, American Chemical Society.

Chen and co-workers [69] were among the first to demonstrate the possibility of conferring multifunctional features (namely, flame retardancy, self-healing features, and superhydrophobicity) to cotton fabrics, through the sequential deposition of an intumescent tri-layered assembly made of branched poly(ethylenimine), ammonium polyphosphate, and fluorinated-decyl polyhedral oligomeric silsesquioxane. In particular, depositing a single tri-layer was enough to achieve self-extinction in vertical flame spread tests (Figure 7); in addition, the presence of the fluorinated-decyl polyhedral oligomeric silsesquioxane accounted for both self-healing (because of the humidity-driven migration of the nanofiller) and superhydrophobic properties (supported by water contact angles as high as 160°). Finally, these multifunctional features were maintained even after more than 1000 cycles of abrasion under a pressure of 44.8 kPa, thus highlighting the big potential of the proposed surface treatment for the design of advanced multifunctional textile materials.

In a research effort toward the design of more environmentally friendly LbL architectures, Pan et al. [70] combined alginate with MgAl hydrotalcites in bi-layered architectures. A total of 20 bi-layered assemblies deposited on cotton accounted for about a 35 and 26% decrease in the peak of the heat release rate and total heat release, respectively, compared with the control fabric. Again, these findings were ascribed to the effective barrier effect toward heat and mass transfer provided by the ceramic nanofiller during the combustion process.

A similar approach was then adopted by Ur Rehman and co-workers [71], who coated cotton with 5, 7, 10, and 15 hybrid bi-layers made of potato starch (positively charged) and vermiculite/titania (negatively charged). Probably due to its high compactness, the LbL coating consisting of seven bi-layers was the best-performing in pyrolysis–combustion flow calorimetry tests, accounting for a significant decrease in both heat release capacity (−25%) and the peak of the heat release rate (−20%) with respect to the untreated control fabric.

Finally, Li and co-workers [72] exploited the LbL method combining phytic acid (a naturally occurring molecule that can be recovered from different plant tissues, like soybeans, oil seeds, and cereal grains [73]) with polyethyleneimine containing silica nanoparticles in assemblies made of two, four, or seven bi-layers. Irrespective of the number of deposited bi-layers, all the LbL-treated fabrics achieved self-extinction in vertical flame spread tests. Further, using the highest number of bi-layers accounted for an important increase in the fabric limiting oxygen index (from 18, untreated cotton, to about 34%) and a decrease in both the peak of the heat release rate and total heat release (by about 75 and 52%, respectively) compared with the control sample. It is worth highlighting that the proposed LbL treatment did not affect the “soft touch” (i.e., the overall mechanical behavior) of the cotton.

## 4. Nanostructured LbL Assemblies for Flame-Retarded Cotton: Latest Research Outcomes

As mentioned in the Introduction paragraph, this part will be specifically devoted to summarizing the most recent research outcomes of flame-retardant nanostructured LbL architectures applied to cotton fabrics.

Gao and co-workers [74] succeeded in fabricating superhydrophobic, flame-retardant, and electrically conductive cotton fabrics. For this purpose, they exploited dipping-assisted LbL, alternately depositing branched polyethyleneimine-modified halloysite nanotubes and phytic acid on the cellulosic substrate, for up to eight bi-layered assemblies. Then, a layer made of octadecyl amine-modified carboxylated carbon nanotube/polydimethylsiloxane was sprayed on the resulting assembly. Irrespective of the number of deposited bi-layers, all the LbL-treated fabrics achieved self-extinction in vertical flame spread tests. Additionally, thanks to the top polydimethylsiloxane-based layer, the surface of the fabrics became highly hydrophobic, with a water contact angle as high as 162°. Finally, the designed systems exhibited piezoresistive properties, with a very stable resistance signal and good reproducibility: these findings suggested their suitability as sensors for detecting human motion, not only in dry environments but also under wet conditions.

**Figure 8:**
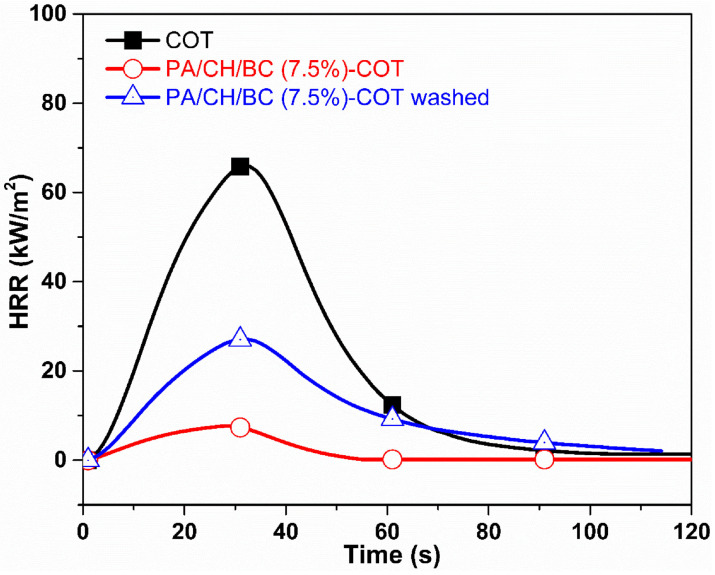
Heat release rate (HRR) vs. time curves for untreated cotton (COT) and for the fabric treated with 5 bi-layers of phytic acid and chitosan embedding biochar at 7.5 wt.%, before (PA/CH/BC 7.5%-COT) and after 10 laundry cycles (PA/CH/BC 7.5%-COT washed). Reprinted with permission from [75]. Copyright 2022, Elsevier.

The possibility of utilizing nanostructured LbL architectures with very low environmental impact, good flame-retardant features, and washing fastness was demonstrated by Cheng and co-workers [75], who deposited up to five bi-layers made of phytic acid and chitosan embedding biochar on cotton. Biochar is a carbon-rich porous material [76], which was obtained through the pyrolysis of rice husks. As assessed by forced-combustion tests (35 kW/m^2^ irradiative heat flux), compared with the control fabric, the deposition of five bi-layers containing 7.5 wt.% of biochar accounted for a remarkable decrease in the peak of the heat release rate and in the total heat release (by about 89% for both), as well as for an important increase in the final residue (around 91%, vs. 0% for the untreated cotton). Additionally, these fabrics achieved a limiting oxygen index as high as 64.1% (vs. 18.6% for the untreated counterparts). Finally, the treated fabrics, subjected to ten washing cycles, highlighted a certain durability, retaining about 60% of their flame retardancy, as shown in Figure 8.

Kang et al. [77] designed tri-layered assemblies made of polyethyleneimine, attapulgite clay, and phytic acid; two, five, or eight tri-layers were LbL-deposited on cotton fabrics. Notwithstanding that, irrespective of the deposited layers, self-extinction was not achieved in vertical flame spread tests; the fabrics treated with the highest number of tri-layers showed a 27% limiting oxygen index value (i.e., much higher than that of the control fabric—17.2%). Conversely, in forced-combustion tests performed at 35 kW/m^2^ irradiative heat flux, a significant decrease in the peak of the heat release rate (around −25%) compared to the untreated fabric was observed, hence demonstrating the flame-retardant effectiveness of the proposed LbL architectures. Finally, the three constituents of the LbL architectures were found active in both condensed and gas phases, either favoring the formation of a carbonaceous–ceramic protective char or exerting a dilution effect on the flammable gases.

**Figure 9:**
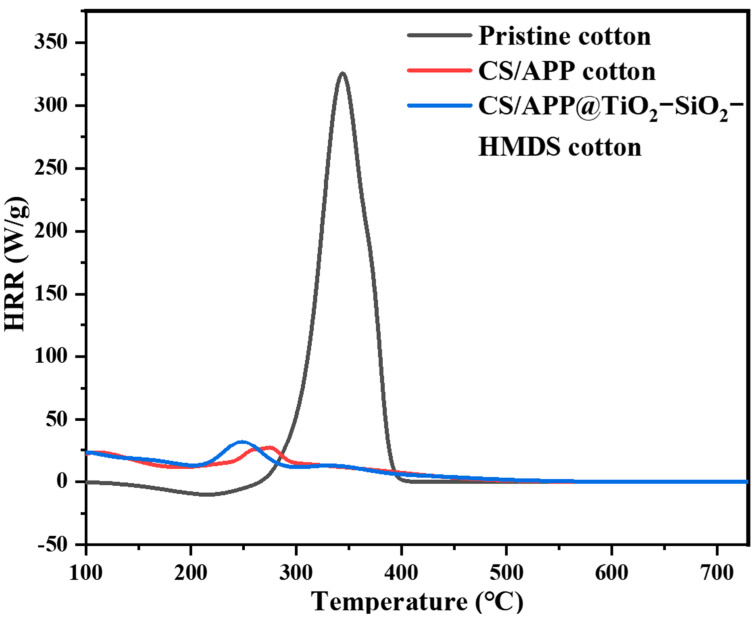
The results from pyrolysis–combustion flow calorimetry tests: heat release rate (HRR) vs. temperature curves for untreated cotton, for the fabric treated with 5 bi-layers of chitosan/ammonium polyphosphate (CS/APP cotton) and for the fabric treated with 5 bi-layers of chitosan/ammonium polyphosphate and silica/titania hexamethyldisilamine sol (CS/APP@TiO_2_-SiO_2_ HMDS cotton) as the top layer. Reprinted from [78] under CC-BY License.

The possibility of designing a single tri-layered assembly capable of providing cotton fabrics with enhanced flame-retardant features, UV-shielding, and superhydrophobicity was recently demonstrated by Li and co-workers [78]. To this aim, the cellulosic material was dipped first in a chitosan solution (at 0.75 wt.%), then in an ammonium polyphosphate bath (at 1.5 wt.%), repeating the dipping steps until five bi-layers were deposited. Finally, the so-treated cotton was immersed in a hexamethyldisilamine sol containing silica and titania nanoparticles and subsequently dried in an oven at 80 °C. The deposited assembly allowed the fabrics to achieve self-extinction in vertical flame spread tests. Additionally, the thermal shielding effect provided by the deposited coating was confirmed by pyrolysis–combustion flow calorimetry tests (Figure 9), which highlighted an impressive decrease in heat release capacity (−90%), the peak of the heat release rate (−91%), and the total heat release (−56%).

Coupling tannic acid-modified MXene nanosheets (which are considered a promising candidate for the development of the next generation of electromagnetic interference shielding materials, thanks to their high metallic electrical conductivity, intercalation ability, large aspect ratio, and tunable surface functional groups [79]) with P, N-co-doped cellulose nanocrystals synthesized on purpose in LbL assemblies allowed for designing multifunctional cotton fabrics that exhibited both interesting flame-retardant features and high electromagnetic shielding [80]. In particular, 10, 15, and 20 bi-layers were deposited on the cellulosic substrate.

**Figure 10:**
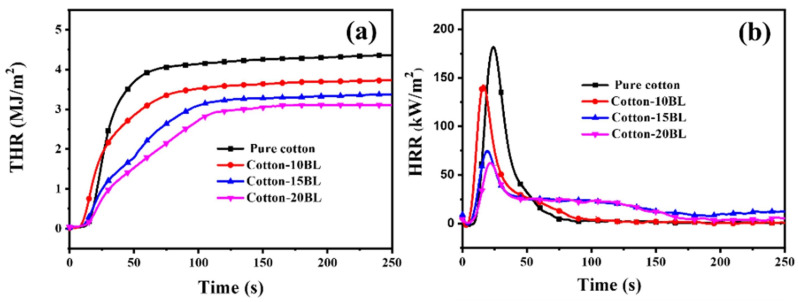
Heat release rate (HRR, (**a**)) and total heat release (THR, (**b**)) vs. time curves for untreated (pure cotton) and LbL-treated cotton (Cotton-XXBL, where XX stands for the number of deposited bi-layers). Reprinted with permission from [80]. Copyright 2022, Elsevier.

The limiting oxygen index values were found to significantly increase with increasing the number of deposited bi-layers, achieving a maximum of 32% for the assemblies containing 20 bi-layers. Additionally, a similar trend was observed in forced-combustion tests (performed at 35 kW/m^2^ irradiative heat flux): 20 bi-layered assemblies accounted for a remarkable lowering of both thermal (total heat release: −31%; peak of heat release rate: −64%) and smoke parameters (Total Smoke Production: −98%), compared with the untreated fabric (Figure 10).

**Figure 11:**
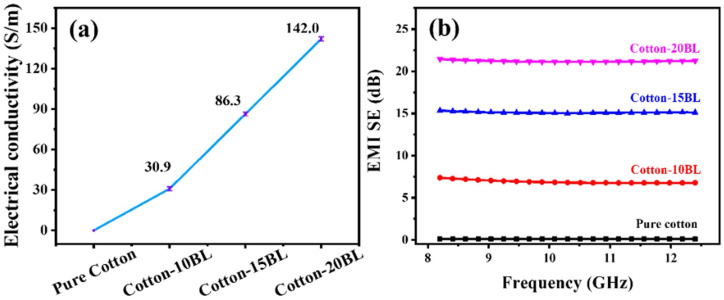
Electrical conductivity (**a**) and electromagnetic interference shielding efficiency (EMI SE, (**b**)) for untreated (pure cotton) and LbL-treated cotton (Cotton-XXBL, where XX stands for the number of deposited bi-layers). Reprinted with permission from [80]. Copyright 2022, Elsevier.

In addition, the LbL-treated cotton fabrics exhibited an increasing electromagnetic interference shielding effect by increasing the number of deposited bi-layers, achieving 21 dB and dissipating 99.2% of the electromagnetic waves in the presence of 20 bi-layers (Figure 11).

Following a similar approach, Zeng and co-workers [81] demonstrated the possibility of designing multifunctional flame-retardant and piezoresistive temperature sensors through the LbL deposition of alternated 2-ureido-4[1H]-pyrimidinone-containing cellulose/Ti_3_C_2_T_x_ MXene nanosheets and montmorillonite/2-ureido-4[1H]-pyrimidinone-containing cellulose bi-layers on cotton. Eight bi-layers accounted for the achievement of a V-0 rating in vertical flame spread tests, together with a limiting oxygen index as high as 40%. In addition, the deposited nanostructured coatings provided an interesting temperature-sensing behavior to the underlying fabric, as well as a fire warning ability (in less than 4 s from the application of a flame), due to the high conductivity and exceptional thermoelectric features of MXene nanosheets. Finally, thanks to extended hydrogen bonding interactions of 2-ureido-4[1H]-pyrimidinone-containing cellulose, the LbL assemblies exhibited self-healing properties, suggesting their potential use for advanced smart firefighting applications.

**Figure 12:**
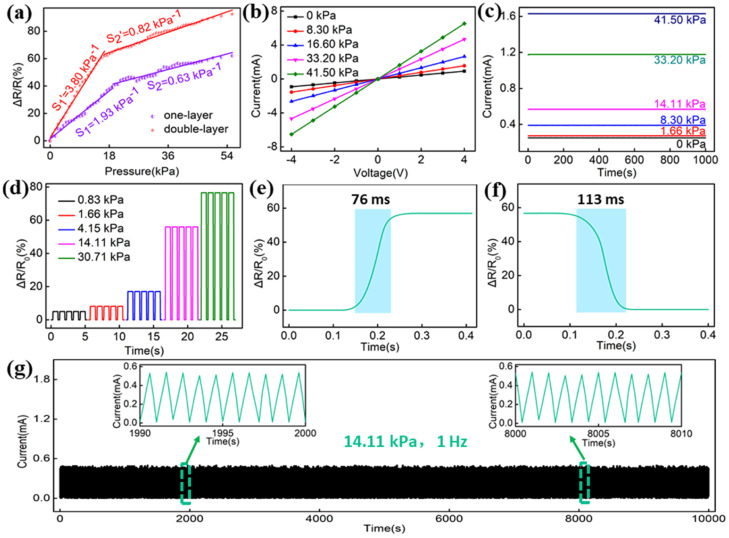
Sensing performances of the LbL-coated fabrics. Sensitivity fitting diagrams of the assemblies made of 1 or 2 bi-layers (**a**). I–V curves (**b**) and stability tests of 2 bi-layered assemblies (**c**). Output currents of the 2 bi-layered assemblies under different external pressures (**d**). The transient response time (**e**) and relaxation time (**f**) of the 2 bi-layered assemblies. Durability tests of the 2 bi-layered assemblies during 10,000 s (**g**). Reprinted with permission from [82]. Copyright 2023, Elsevier.

In a further research effort, Zhu and co-workers [82] designed a highly sensitive piezoresistive flame-retardant sensor by alternately depositing amino-functionalized carbon nanotubes and ammonium polyphosphate (one or two bi-layers) on cotton. The so-obtained assemblies showed self-extinction in vertical flame spread tests, as well as a limiting oxygen index value of 37.6% (for two deposited bi-layers, vs. 18.4% for the control fabric). Interestingly, the two deposited bi-layered assembly was able to trigger the fire-alarming system within just 2 s. Finally, as shown in Figure 12, the overall performance of the designed piezoelectric assemblies was satisfactory, highlighting a good sensitivity of 3.8 kPa^−1^ over the pressure range of 0–16.62 kPa, good electrical stability, and even durability.

Lama and co-workers [83] deposited up to six bi-layers made of polyethyleneimine (positively charged) and halloysite clay nanotubes (negatively charged) on cotton. The deposition of just two bi-layers of the hybrid architecture accounted for self-extinction, as assessed by vertical flame spread tests. Additionally, the possibility of incorporating chloramphenicol (i.e., an antimicrobial additive against *S. Marcescens* bacterium) in the halloysite clay nanotubes of the LbL assembly provided the treated cotton with enhanced antibacterial activity, increasing the inhibition zone around the fabric.

The concurrent presence of flame retardant and antibacterial properties in LbL assemblies on cotton was further envisaged by Attia and co-workers [84], who deposited 3, 5, and 10 bi-layers made of rennet casein nanoparticles (in anionic form; average size: 116 nm) and chitosan nanoparticles (in cationic form; average size: 26 nm), hence designing fully bio-based assemblies. The deposition of at least five bi-layers accounted for self-extinction without afterglow phenomena, an important suppression of toxic gases, and a 29.5% limiting oxygen index (vs. 18.5% for the control fabric). Finally, the designed assemblies were responsible for the limited growth of *E. coli* bacteria, highlighting an inhibition zone of 16 mm.

A nice example that combines the LbL method with the sol–gel approach was proposed by Zhang et al. [85], who deposited 5 or 10 bi-layers made of gelatin (positively charged) and adenosine 5′-monophosphate (negatively charged). The resulting assemblies were finally immersed into a silica sol bath, to obtain the formation of a silica gel top layer. As revealed by vertical flame spread tests, the 10 bi-layered assemblies coated by the silica gel layer were self-extinguishing; compared with untreated cotton, these systems also showed a remarkable decrease in both the peak of the heat release rate (about −50%) and total heat release (around −19%). Finally, the silica gel coating was a key component for enhancing the washing fastness of the proposed assemblies.

B,N-co-doped carbon dots were dispersed in alginate and alternately deposited together with polyethyleneimine on cotton fabrics [86], designing assemblies made of 4, 8, and 12 bi-layers. The presence of carbon dots not only conferred photoluminescent features to the cellulosic substrate when exposed to 365 nm UV irradiation but also UV-shielding properties, with average ultraviolet protection factors that significantly increased with increasing the number of deposited bi-layers, approaching 64.1 for the 12 bi-layer-treated fabric. Additionally, the treated fabrics, irrespective of the number of deposited bi-layers, did not reach self-extinction in vertical flame spread tests but showed a decrease in the peak of the heat release rate during pyrolysis–combustion flow calorimetry tests (achieving −15% for 12 bi-layered assemblies, compared with the control fabric). The UV-shielding and flame-retardant performance of these assemblies was further improved in a more recent paper published by the same research group [87].

The use of carbon nano-objects for the design of efficient LbL flame-retardant architectures for cotton was thoroughly investigated by Xu and co-workers [88], who designed tri-layered spray-assisted architectures made of 3-aminopropyl triethoxysilane, modified single-walled carbon nanohorns, and ammonium polyphosphate on cotton fabrics. In particular, four tri-layered assemblies allowed for achieving self-extinction in vertical flame spread tests (fabric’s damage length: 3.9 cm) and for considerably decreasing both the total heat release (by about 57%) and peak of the heat release rate (by around 93%), compared to the control fabric. These findings were attributed to the formation of a stable, protective, and coherent P-N-Si heterocyclic char layer on the surface of the cellulosic substrate during the combustion process.

Interesting flame-retardant and antibacterial features were provided to cotton fabrics by first dip-coating five bi-layers of polyethyleneimine/ammonium polyphosphate and subsequently immersing the treated fabrics in a silver–phosphoryl triazole complex solution [89]. The so-obtained fabrics reached self-extinction (with 0 s of both afterflame and afterglow times) in vertical flame spread tests and achieved a 33.5% limiting oxygen index. Further, they showed remarkably enhanced thermal parameters in forced-combustion tests performed at 35 kW/m^2^, with an important decrease in the peak of the heat release rate (−91%) and total heat release (−78%), and a very high residue at the end of the tests (35.1% vs. 6.6% for the untreated fabric). Finally, the LbL assembly was very effective in inhibiting the growth of both *S. aureus* and *E. coli*, highlighting inhibition rates as high as 95% for both bacteria: this finding was ascribed to the high antimicrobial activity of the silver ions embedded in the assembly.

**Figure 13:**
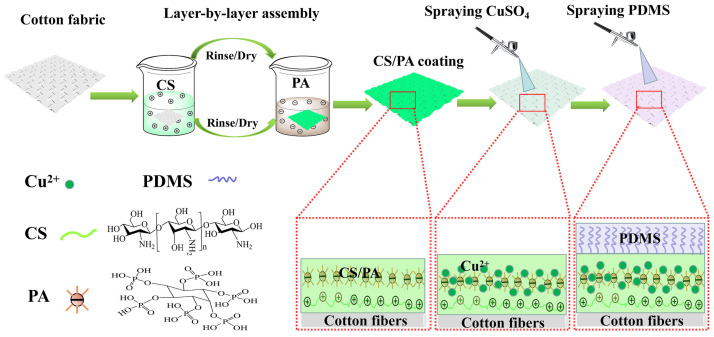
A scheme of the construction process for the design of LbL multifunctional coatings on cotton. Legend: CS = chitosan; PA = phytic acid; PDMS = poly(dimethyl siloxane). Reprinted with permission from [90]. Copyright 2024, Elsevier.

Very recently, Fang et al. [90] demonstrated high multifunctionality (specifically referring to flame retardancy, superhydrophobicity, antibacterial activity, and durability—in terms of washing fastness and abrasion resistance), which can be achieved by depositing four bi-layered phytic acid/chitosan assemblies on cotton fabrics and by further spraying divalent copper ions (from a CuSO_4_ solution) and a final poly(dimethyl siloxane) top layer, as schematized in Figure 13. The so-obtained fabrics achieved self-extinction in vertical flame spread tests and showed a remarkable decrease in the peak of the heat release rate (−77.8%), total heat release (−53.5%), and Total Smoke Production (−75.2%), compared to the control fabric, in forced-combustion tests (carried out at 35 kW/m^2^ irradiative heat flux). Further, the presence of the poly(dimethyl siloxane) top layer accounted for water contact angles beyond 150°. Thanks to the presence of copper ions, the deposited assemblies showed antibacterial rates against *S. aureus* and *E. coli* beyond 99%. Very interestingly, all these multifunctional features were substantially maintained after either laundry cycles or abrasion resistance tests, hence indicating the high durability of the designed LbL architectures.

## 5. Conclusions and Perspectives

The present review has tried to summarize the current state of the art of the use of nanostructured LbL architectures, capable of conferring flame-retardant features to cotton. Undoubtedly, nanostructuring the surface of this cellulosic substrate through the layer-by-layer approach is a valuable, reliable, and effective method in flame retardancy, thanks to the wide (and practically unlimited) availability of the layers’ constituents and the tunability of the designed and obtained nanostructures. Further, the possibility of projecting LbL treatments that can confer multifunctional features to cotton (such as hydrophobicity, self-healing ability, antimicrobial activity, and flame retardancy, among others) makes this surface-engineered approach even more appealing. Specifically referring to flame retardancy, LbL assemblies often account for thermal shielding and/or intumescent effects, based on the chemical nature and composition of the deposited layers, favoring, when possible, the occurrence of synergistic effects among the components.

Textile materials are quite challenging when they need to be flame-retarded, because of their irregular surface and the limited possibility to incorporate effective flame retardants directly into their bulk (this is, indeed, possible only for some synthetic fibers/fabrics, exploiting melt compounding and subsequent spinning). In this regard, the LbL strategy surely shows a big potential and deserves further investigation, also considering that the conventional FRs, though very effective, sometimes face limitations in use, because of possible toxicity issues and/or limited eco-sustainability [91,92]. Conversely, the identification and selection of bio-based components for the layer-by-layer architectures greatly support the up-to-date circular economy approach [93,94,95].

However, the LbL strategy is far from being fully exploited for several reasons. First, this surface-engineered method was born basically for lab-scale research activities, and its scalability to larger manufacturing processes, despite some nice attempts [96,97], is still a challenging issue and very difficult to solve. The LbL strategy based on the dipping technique is boring, time-consuming, and requires significant manual “exercise”, despite the possibility of purchasing and employing automatic robots. On the other hand, spraying LbL seems more suitable for potential exploitation at a pilot/industrial scale: indeed, it is faster than dipping and impedes the cross-contamination of the utilized baths. The second (and not less important) issue regarding LbL is the overall durability of the applied assemblies, not only in terms of resistance to laundry cycles but also to physical friction and bacterial erosion. Most of the layer-by-layer assemblies deposited on cotton are based on electrostatic interactions (neither too weak nor too strong), which take place among the layers. Therefore, as it happens during a laundry occasion, putting the treated fabrics in contact with an aqueous solution containing surfactants, at a certain temperature and for a certain time, usually results in a (at least) partial removal of the deposited assembly, hence an overall worsening of the flame-retardant features. Generally speaking, it is thus demanded to strongly enhance the overall “endurance” of the LbL-treated cotton: this can be fulfilled by replacing the electrostatic interactions with stronger covalent bonds (i.e., hydrolytically stable bonds, particularly C–C bonds) or by depositing LbL assemblies possessing superhydrophobic features.

It may be expected, in the forthcoming years, that the integration of specific manufacturing procedures into the LbL technique can lead to a significant reduction in the process times, making this strategy closer to potential market exploitation.

In addition, it would be necessary to perfect suitable and reliable strategies for predicting the specific features and the overall performance of nanostructured flame-retardant LbL architectures, even before their deposition on cotton (or, more generally, on any other substrate to treat), exploiting the direct knowledge on the adopted experimental parameters (such as the chemical structure of the LbL constituents, the assembly method, and the deposition conditions). Indeed, these predictive models may contribute to decreasing the current gap between layer-by-layer design and its market/industrial exploitation.

## Data Availability

No new data were created or analyzed in this study. Data sharing is not applicable to this article.
